# Targeting CYP2J2 to Enhance the Anti-Glioma Efficacy of Cannabinoid Receptor 2 Stimulation by Inhibiting the Pro-Angiogenesis Function of M2 Microglia

**DOI:** 10.3389/fonc.2020.574277

**Published:** 2020-11-27

**Authors:** Xuejiao Lei, Xuezhu Chen, Yulian Quan, Yihao Tao, Junlong Li

**Affiliations:** ^1^Department of Neurosurgery and Key Laboratory of Neurotrauma, Southwest Hospital, Third Military Medical University (Army Medical University), Chongqing, China; ^2^Department of Neurosurgery, The Second Affiliated Hospital, Chongqing Medical University, Chongqing, China; ^3^Office of Scientific Research Administration, Southwest Hospital, Third Military Medical University (Army Medical University), Chongqing, China

**Keywords:** glioma, JWH133, M2 microglia, CYP2J2, 11,12-EET, angiogenesis

## Abstract

Enhancing the therapeutic efficacy of anti-tumor drugs is essential for cancer management. Although cannabinoid receptor 2 (CB2R) stimulation exerts anti-tumor action in glioma cells by regulating cellular proliferation, differentiation, or apoptosis, selective CB2R agonist alone does not achieve a satisfactory therapeutic outcome. Herein, we aimed to evaluate the possible strategy for enhancing the anti-glioma efficacy of JWH133, a selective CB2R agonist. In this study, immunofluorescence and qRT-PCR were used to investigate microglia polarization. Tumor growth was monitored *via* bioluminescent imaging using the IVIS Spectrum System. The angiogenesis of human brain microvascular endothelial cells (HBMECs) was detected by the tube formation assay. qRT-PCR was used to investigate cytochrome P450 2J2 (CYP2J2) and 11,12-epoxyeicosatrienoic acid (11,12-EET) expression. Our results showed that administration of JWH133 significantly promoted microglial M2 polarization both *in vitro* and *in vivo*. The medium supernatant of M2 microglia induced by JWH133 treatment facilitated angiogenesis of HBMECs. CYP2J2 expression and 11,12-EET release in the supernatant of JWH133-induced M2 microglia were significantly upregulated. Treatment with 11,12-EET prompted HBMEC angiogenesis and glioma growth. CYP2J2 knockdown restrained the release of 11,12-EET and significantly enhanced the anti-tumor effect of JWH133 on glioma. This study showed that targeting CYP2J2 might be a beneficial strategy to enhance the anti-glioma efficacy of JWH133 by inhibiting the pro-angiogenesis function of M2 microglia.

## Introduction

Cannabinoids, the active components of *Cannabis sativa* and their derivatives, are known to exert their effects on a wide spectrum of diseases such as nervous system diseases, glaucoma, asthma, cardiovascular diseases, and tumors (1, 2). To date, two endocannabinoid receptors have been cloned *in vitro* and widely studied: cannabinoid receptor-1 (CB1R) and CB2R (3, 4). In the central nervous system (CNS), CB1R is mainly distributed in the presynaptic membranes of nerve terminals and regulates the release of neurotransmitters to mediate cannabinoid psychoactivity (5). However, CB2R, mainly located in the glial cells, is not known to mediate psychoactivity but plays an important role in immune regulation and inhibition of cytokine release (6, 7), which has been well documented in treating neurological diseases such as neurodegeneration disorders (8), multiple sclerosis (9), stroke (10), and spinal cord injury (11) by modulating microglial activities. Furthermore, CB2R is also expressed in tumor cells. Different types of tumor cells can over-express CB2R and are as such very attractive for cancer treatment (2). A previous study showed that the extent of CB2R expression was directly related with glioma malignancy (12). Interestingly, local administration of the selective CB2 agonist—JWH133 resulted in considerable regression of glioma by inducing glioma cell death (12) and suppressing the production of vascular endothelial growth factor (VEGF) in glioma cells (13). Various non-neoplastic cell types are present in the microenvironment of gliomas, and 30–50% of the glioma cells are microglia or macrophages (14). However, little is known about the effect of CB2 stimulation on microglia/macrophages in glioma.

Microglia are a type of resident macrophages that colonize the brain early during development. Microglia and non-parenchymal macrophages in the brain are mononuclear phagocytes that are increasingly recognized to be essential players in stroke, spinal cord injury (SCI), and brain cancers (15, 16). Microglia/macrophages have multidimensional activation states in CNS diseases and injuries, and these states can be identified by specific markers. Although different microglia/macrophage classification schemes are found based on current single cell sequence analyses in different diseases (17), the main activation states are described as follows: the activated (M1) phenotype that is mediated by pro-inflammatory processes and can be characterized by the expression of CD68, CD86, and iNOS, and the alternatively activated (M2) phenotype that is mediated by anti-inflammatory processes and can be identified by the expression of CD206, Ym1, and arginase-1 (18). In CNS injury including stroke, traumatic brain injury (TBI), and SCI, the M1 phenotypic cells aggravate tissue damage and impede the repair process by releasing destructive proinflammatory mediators. On the contrary, M2 phenotypic cells promote brain recovery by clearing cell debris, resolving local inflammation, and releasing a plethora of trophic factors (10). Therefore, many studies have indicated that strategies based on promoting M1 to M2 phenotypic conversion of microglia/macrophages might provide therapeutic potential for the aforementioned diseases. However, in brain malignant gliomas, the M1 phenotypic microglia and tumor-associated macrophages (TAMs) have been reported to inhibit glioma tumor growth by presenting antigens to adaptive immune cells, producing pro-inflammatory cytokines, and phagocytosing tumor cells (19). High expression of M1 marker was positively correlated with increased patient survival (CD74) (20). On the other hand, alternative M2 phenotype microglia and TAMs promoted glioma growth/survival by secreting proangiogenic factors and immunosuppressive cytokines. Increased proportion of M2 phenotype microglia and TAMs has been associated with poor prognosis in gliomas (14, 21). Therefore, converting M2 to M1 phenotype is suggested as a potential therapeutic strategy to reduce glioma growth. Furthermore, our studies have also shown that JWH133 treatment alleviated brain injury by promoting the microglia/macrophage polarization from pro-inflammatory M1 to anti-inflammatory M2 phenotypic conversion in a stroke model (10). However, this JWH133-induced pro-M2 polarization effect could likely weaken its anti-glioma function (12), and the molecular mechanisms underlying the pro-tumorigenic functions of M2 microglia/TAMs after JWH133 treatment remain to be elucidated.

Epoxyeicosatrienoic acids (EETs), the cytochrome P450 (CYP) epoxygenases-derived metabolites of arachidonic acid, exert a critical role in vascular functions by regulating the membrane hyperpolarization of smooth muscle cells (22). A recent study revealed that EETs induced the expression of VEGF and promoted angiogenesis in endothelial cells (ECs) (23). Additionally, studies reported that the cytochrome P450 2J2 (CYP2J2) is overexpressed in human cancer tissues. Overexpression of CYP2J2 and addition of synthetic EETs could enhance tumor growth *via* promoting angiogenesis (24, 25). In addition, increased expression of arachidonic acid was found in reactive microglia (26), and the expression of microglial CYPs increases under inflammatory conditions (27), thereby indicating more EETs will be released from reactive microglia/macrophages. It is therefore very interesting to investigate whether and which phenotype of microglia/macrophage participates in the above metabolic process in gliomas.

Herein, we hypothesized that JWH133 treatment can exert anti-glioma action, concurrently leading to microglial M2 polarization in glioma, which promotes glioma growth *via* enhancing angiogenesis by increasing the expression of CYP2J2 and 11,12-EET, an isomer of EETs. Therefore, inhibiting the pro-angiogenesis function of M2 microglia probably enhances the anti-glioma effect of JWH133. We believe this study provides a novel mechanism and strategy for enhancing CB2R-based anti-glioma therapies.

## Materials and Methods

### Animals

Male nude mice aged 4–6 weeks were purchased from the Experimental Animal Center at the Third Military Medical University and maintained in the Laboratory Animal Centre at Southwest Hospital (Gaotanyan Street No.30, Shapingba District, Chongqing, China). No randomization was used for the *in vivo* experiments. The mice were housed in a temperature-controlled room in specific-pathogen-free conditions under a standard 12-h light/dark cycle, with *ad libitum* access to food and water. All experiments are reported in compliance with the Animal Research: Reporting *in Vivo* Experiments (ARRIVE) guidelines. The experimental protocols were approved by the Ethics Committee of the Third Military Medical University and performed according to the Guide for the Care and Use of Laboratory Animals.

### U87 Cell Culture and Implantation

The U87 glioma cell line was purchased from American Type Culture Collection and maintained in DMEM supplemented with 10% fetal bovine serum (FBS) (Gibco). The lentiviral vector with firefly luciferase plasmid was stably transfected into U87 cells (U87-Luc). Mice (n = 5 per group) were deeply anesthetized by intraperitoneal injection of 2 mg ketamine and 0.4 mg xylazine in 0.9% saline. Intracranial glioma xenografts were established in nude mice by stereotactic injection of 10^4^ U87-Luc cells in 5 μl Hank’s buffer into the right striatum (bregma coordinates: 0.8 mm anterior and 2 mm lateral to the midline; depth, 3 mm). JWH133 (1.0 mg/kg, Tocris Bioscience, Catalog#: 1343); AM630 (1.0 mg/kg, Tocris Bioscience, Catalog#: 1120); and 11,12-EET (50 μg/kg, Cayman Chemicals, Catalog#: 50511) were injected intraperitoneally on the seventh day after tumor implantation for 7 days. Further, lentiviral plasmid expressing CYP2J2 shRNA and control non-targeting shRNA were constructed by Shanghai OBIO Technology Co., Ltd. The viruses (2 μl, 10^9^ copies/ml) were injected stereotactically at the same location of tumor implantation on the seventh day after tumor implantation. Two weeks after transplantation, tumor-bearing mice were tested for live imaging. Tumor growth was monitored *via* bioluminescent imaging using the IVIS Spectrum System and quantified by Living Image Software.

### Microglia Culture

Microglial cells were isolated from the brain cortex as described in previous studies (18, 28). Briefly, the cortex of mice at postnatal day 2 was dissected under an anatomical microscope after perfusion with ice-cold PBS. After stripping the cerebral pia mater, the tissue was enzymatically digested using Neural Tissue Dissociation Kit (Miltenyi Biotec, Germany) for 35 min at 37°C. Then, the digested debris was filtered using a 40-μm cell strainer. After myelin removal, cells were stained with PE-conjugated anti-CD11b antibodies (Miltenyi Biotec) in IMAG buffer (PBS supplemented with 0.5% BSA and 2 mM EDTA) for 10 min followed by incubation for 15 min with anti-PE magnetic beads. CD11b^+^ cells were separated in a magnetic field using MS columns (Miltenyi Biotec). Then, a cell suspension was prepared in DMEM/F12 containing 1% double antibodies and 10% FBS, placed into a 5% CO_2_ incubator and incubated at 37°C for 12 h; the suspension was replaced after the cells adhered to the walls. Thereafter, the culture medium was replaced once every three days. The microglia cell purity was more than 95% as determined by Iba1 staining. The total time for isolation was less than 2 h.

### Polarization of Microglia

Purified microglia were spread on a six-well plate at a concentration of 10^5^/well and cultured for 72 h. Then, serum-free culture medium was used to induce M0 microglia; serum-free culture medium containing lipopolysaccharides (LPS, 100 ng/ml; Sigma, Catalog#: L2630) + IFN-*γ* (25 μg/ml; Sigma, Catalog#: IFN-*γ*) was used to induce M1 microglia (18); and serum-free culture medium containing CB2R agonist JWH133 (4 μM) was used to induce M2 microglia (10). The microglia were cultured under the three above-mentioned culture media conditions for 24 h, after which the media were replaced, and then serum-free culture media were added to continue the culturing. After 24 h, 1% FBS was added to the collected supernatants, and the culture media were used to treat human brain microvascular endothelial cells (HBMECs). Furthermore, the primary microglia were treated with AM630 (1 μM) and ERK antagonist SCH772987 (1 μM, MedChemExpress, Catalog#: HY-50846) for 24 h to explore the mechanism. Additionally, the 11,12-EET level was analyzed by LC-MS/MS, as previously described (29).

### Endothelial Tube Formation Assay

First, 50 μl of Matrigel (BD BiocoatTM, Catalog#: 356234) was placed in each well of a 96-well plate and incubated at 37°C for 30 min. The digested HBMECs were counted by a cell counter. Then, 50 μl of cell suspension containing 1 × 10^4^ cells were added to each Matrigel-treated well and maintained at 37°C for 3 h. Then, the original medium was replaced with the corresponding M0, M1, and M2 microglia culture supernatants and the medium containing 11,12-EET (2 μM, resuspended in DMSO with original organic solvent evaporated). Next, the HBMECs were transferred with lentiviral plasmid expressing CYP2J2 shRNA and control non-targeting shRNA. After 12 h of culture, the results were observed under a microscope and photographed.

### Quantitative Real-Time PCR

qRT-PCR analysis was performed as previously described (30). Briefly, total RNA was extracted with Trizol Reagent (Invitrogen), and cDNA was synthesized using a reverse transcription kit (TaKaRa). The target gene mRNA level was measured using SYBR Premix Ex Taq II (TaKaRa) and a CFX96 Real-Time PCR Detection System (Bio-Rad). The relative target gene mRNA level was calculated using the 2−ΔΔCt method. *GAPDH* expression was used for normalization. The sequences of primers used in this study are listed in **Table 1**.

**Table 1:** Forward and reverse sequence of the used primers.

Gene	GeneBank	Forward sequence	Reverse sequence
TNFα	NM_013693	GGTGCCTATGTCTCAGCCTCTT	GCCATAGAACTGATGAGAGGGAG
IL1β	NM_008361	TGGACCTTCCAGGATGAGGACA	GTTCATCTCGGAGCCTGTAGTG
IL4	NM_021283	ATCATCGGCATTTTGAACGAGGTC	ACCTTGGAAGCCCTACAGACGA
IL10	NM_010548	CGGGAAGACAATAACTGCACCC	CGGTTAGCAGTATGTTGTCCAGC
CD68	NM_009853	GGCGGTGGAATACAATGTGTCC	AGCAGGTCAAGGTGAACAGCTG
CD86	NM_019388	ACGTATTGGAAGGAGATTACAGCT	TCTGTCAGCGTTACTATCCCGC
iNOS	NM_010927	GAGACAGGGAAGTCTGAAGCAC	CCAGCAGTAGTTGCTCCTCTTC
CD206	NM_008625	GTTCACCTGGAGTGATGGTTCTC	AGGACATGCCAGGGTCACCTTT
Arg1	NM_007482	CATTGGCTTGCGAGACGTAGAC	GCTGAAGGTCTCTTCCATCACC
Ym1	NM_009892	TACTCACTTCCACAGGAGCAGG	CTCCAGTGTAGCCATCCTTAGG
CYP2J2	NM_000775	TGCTGTCATCCATGAGGTGCAG	CGCCGTCAAATTGGTCAGGATC
GAPDH	NM_008084	CATCACTGCCACCCAGAAGACTG	ATGCCAGTGAGCTTCCCGTTCAG

### Immunofluorescence Staining

The paraformaldehyde-fixed glioma tissue or primary microglia slides were incubated with 3% BSA blocking solution for 1 h, then incubated with primary antibody overnight at 4°C and incubated with Alexa Fluor^®^ secondary antibody at room temperature for 2 h as shown previously (31). Then, the images were acquired by confocal microscopy (Carl Zeiss, Weimar, Germany) and examined using Zen 2011 software (Carl Zeiss, Weimar, Germany) after mounting the anti-fluorescent quencher (Santa Cruz Biotechnology, sc-24941, United States). Primary antibodies used for staining included anti-Iba1 (Abcam, ab5076, 1:50); anti-CD68 (BD Biology, MCA1957, 1: 200); and anti-CD206 (BD Biology, MCA223, 1: 200). For each specimen, the gene expression was calculated by examining five randomly selected microscopic fields at 40× magnification (ImageJ).

### Western Blotting

Total protein from the primary microglial cells treated with JWH133 alone and co-treated with JWH133 and AM630 was lysed in ice-cold RIPA (Sigma-Aldrich, St. Louis, MO, United States) supplemented with protease inhibitor cocktail (Roche, Indianapolis, IN, United States) as described in a previous study (32). The protein concentration was determined by an enhanced BCA Protein Assay Kit (Beyotime, Beijing, China). Then, 20 μg of protein per sample was resolved on 10% SDS-PAGE gels and transferred onto PVDF membranes (Roche, Indianapolis, IN, United States). The membranes were first incubated for 2 h at room temperature in TBST containing BSA, 0.05% Tween 20, and incubated by primary antibodies overnight at 4°C against ERK (Abcam, ab184699, 1: 1,000), anti-p-ERK (BioVision, 3441-100, 1:1,000), and anti-CYP2J (Abcam, ab151996, 1:1,000) followed by incubation with horseradish peroxidase (HRP)-conjugated secondary antibody at room temperature for 1 h. The protein bands were detected by ChemiDocTM XRS C imaging system (Bio-Rad, Berkeley, CA, USA) using the WesternBright ECL Kits (Advansta, Menlo Park, CA, USA). Densitometric measurement of each membrane was performed using Image LabTM software (Bio-Rad).

### MR Imaging

The surviving mice were imaged 14 days after implantation by a Bruker Biospec 7.0 T small animal MRI scanner, with ADVANCE III hardware/software. Tumor volume was measured by T2-weighted images. Standard analysis methods were used to compute the images.

### Statistical Analysis

Statistical analysis was performed using SPSS 18.0 software. Data were expressed as the mean ± SEM. Intergroup comparisons were analyzed using two-tailed Student’s *t*-tests. Other data were analyzed using one-way ANOVA (one factor) followed by Tukey’s *post-hoc* test. P value <0.05 was considered to indicate statistical significance.

## Results

### JWH133 Promoted Microglia M2 Polarization Both *In Vitro* and *In Vivo*

**Figure 1:**
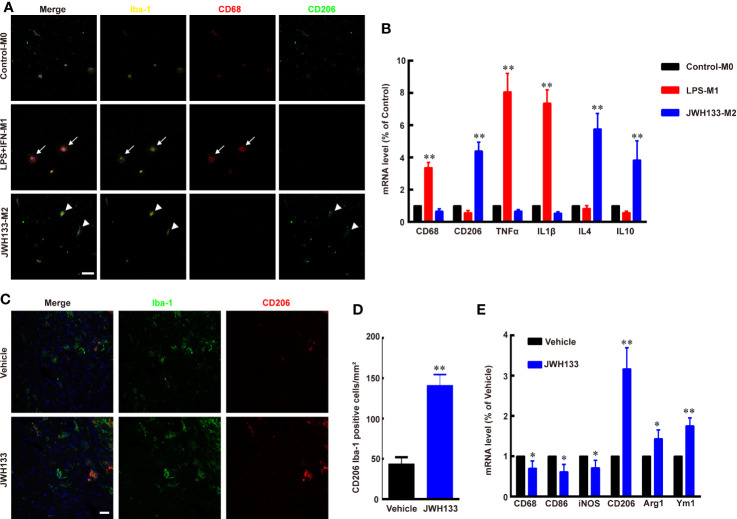
JWH133 promotes microglia/macrophage M2 polarization *in vitro* and *in vivo*. **(A)** Representative primary microglial immunofluorescence staining for Iba-1, CD68, and CD206 in Control, LPS + IFN, and JWH133 treatment groups (white arrows indicate typical M1 microglia, and white arrowheads indicate typical M2 microglia), Scale bar: 50 μm. **(B)** Primary microglial mRNA level (% of Control level) of CD68, CD206, TNFα, IL1β, IL4, and IL10 in the control, LPS + IFN, and JWH133 treatment groups. Data presented as mean ± S.E.M, n = 5; ^**^P < 0.01 indicates significant differences *vs.* Control. One-way ANOVA was performed followed by Tukey’s *post hoc* test. **(C)** Representative immunofluorescence staining for Iba-1 and CD206 of microglia/macrophage around the glioma in Vehicle and JWH133-treated groups *in vivo*. Scale bar: 20 μm. **(D)** Quantitative data of CD206 and Iba-1 double positive cells per mm^2^ around glioma in Vehicle and JWH133- treated groups *in vivo*. Data are shown as mean ± S.E.M, n = 5; ^**^P < 0.01 indicates significant differences *vs.* Vehicle (Student’s *t*-tests). **(E)** mRNA level (% of Vehicle level) of CD68, CD86, iNOS, CD206, Arg1, and Ym1 in Vehicle and JWH133-treated groups *in vivo*. Data are presented as mean ± S.E.M, n = 5; ^*^P < 0.05 and ^**^P < 0.01 indicate significant differences *vs.* Vehicle (Student’s *t*-tests).

To test the function of CB2R agonist JWH133 in microglia/macrophage polarization, we extracted primary microglia from the cortex of newborn mice and cultured them in different culture medium. The Iba1-positive M0 microglia expressed low levels of M1 subtype marker CD68 and M2 subtype marker CD206, as the microglia had not been activated (**Figure 1A**). After being induced by LPS + IFN-*γ*, the microglia became round, and the expression level of CD68 was apparently increased, whereas the expression levels of CD206 were low (**Figure 1A**). Further, the mRNA expression levels of CD68, TNFα, and IL1β also significantly increased in M1 microglia (^**^P < 0.01 *vs.* Control; **Figure 1B**). Consistent with a previous study (10), JWH133 treatment induced M2 typical microglia subtype, which expressed high levels of CD206 and low levels of CD68 (**Figure 1A**). The PCR assays were consistent with this finding that the mRNA expression level of CD206, IL4, and IL10 were significantly increased in M2 microglia (^**^P <0. 01 *vs.* Control; **Figure 1B**). Besides, *in vivo* experiments showed that there were more numbers of CD206+ and Iba1+ co-labeled microglia/macrophages around the glioma in the JWH133-treated group than in Vehicle group (^**^P < 0.01 *vs.* Vehicle; **Figures 1C, D**). JWH133 treatment also significantly increased the mRNA level of M2 subtype markers CD206, Arg1, and Ym1 and decreased the mRNA level of M1 subtype markers CD68, CD86, and iNOS in the tissues around glioma, *in vivo* (^*^P < 0.05 and ^**^P < 0.01 *vs.* Vehicle; **Figure 1E**).

### M2 Microglia Supernatant Induced by JWH133 Promotes Angiogenesis *In Vitro* in HBMECs

**Figure 2:**
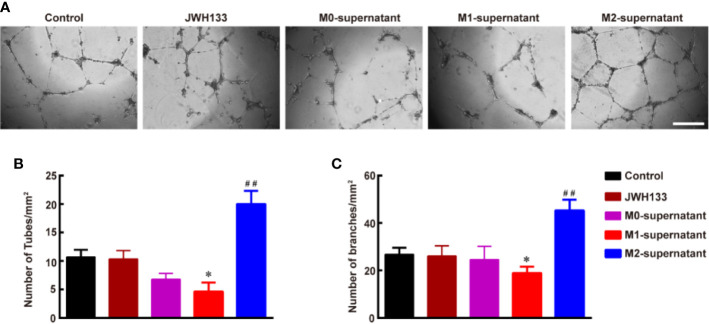
Culture supernatant of M2 microglia induced by JWH133 promotes angiogenesis *in vitro* in HBMECs. **(A)** Representative images of tube formation of HBMECs in the Control, JWH133, M1-culture supernatant, and M2-culture supernatant groups. Scale bar: 200 μm. **(B, C)**, Quantitative data of number of tubes (B, per mm^2^) and branches (C, per mm^2^) in HBMECs in the Control, JWH133, M1-culture supernatant, and M2-culture supernatant groups. Data are presented as mean ± S.E.M, n = 5; ^*^P < 0.05 and ^##^*P* < 0.01 indicate significant differences *vs.* the Control group (one-way ANOVA followed by Tukey’s *post hoc* test).

To understand the effects of different subtypes of microglia on angiogenesis, we used the microglial supernatants and JWH133 to treat HBMECs. We found that individual intervention of JWH133 did not promote angiogenesis of HBMECs. Interestingly, the microglial M2 type supernatant after JWH133 treatment significantly promoted angiogenesis by increasing the number of tubes and branches (^**^P < 0.01 *vs.* Control; **Figures 2A–C**). However, the M1 supernatant induced by LPS + IFN-*γ* inhibited the angiogenesis progression by decreasing the number of tubes and branches (^*^P < 0.05 and ^**^P < 0.01 *vs.* Control; **Figures 2A–C**). These results indicated that there might be some factors in the M2 supernatant capable of promoting angiogenesis of HBMECs.

### JWH133 Treatment Promoted the Creation of EETs of M2 Microglia

**Figure 3:**
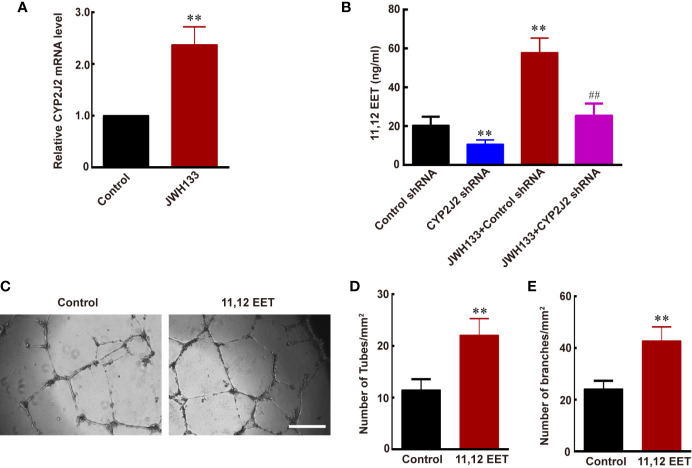
JWH133-induced M2 microglia promotes angiogenesis *via* increasing the expression of CYP2J2/11,12-EET. **(A)** Relative CYP2J2 mRNA level (% of Control level) in the Control and JWH133 groups. **(B)** 11,12-EET content (ng/ml) for Control shRNA, CYP2J2 shRNA, JWH133 + Control shRNA, and JWH133 + CYP2J2 shRNA groups. **(C)** Representative images of tube formation of HBMECs in groups of Control and 11,12-EET. Scale bar: 200 μm. **(D, E)** Quantitative data of number of tubes (**D**, per mm^2^) and branches (**E**, per mm^2^) in HBMECs in the Control and 11,12-EET groups. Data are presented as mean ± S.E.M, n = 5; ^**^P < 0.01 indicates significant differences *vs.* the Control group; ^##^*P* < 0.01 indicates significant differences *vs.* the JWH133 group (one-way ANOVA followed by Tukey’s *post hoc* test).

**Figure 4:**
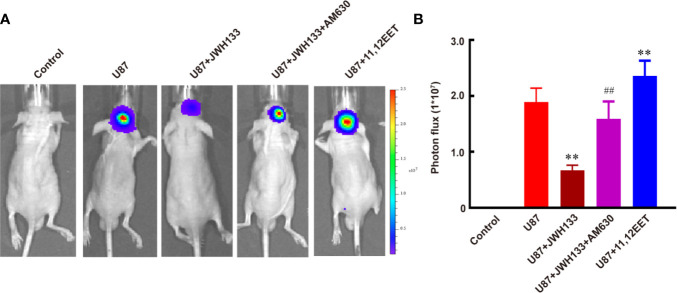
JWH133 treatment inhibits the growth of glioma two weeks after implantation. Representative bioluminescent images **(A)** and the quantification **(B)** of the tumor-bearing mice implanted with U87 in the Control, U87, U87 + JWH133, U87 + JWH133 + AM630, and 11,12-EET groups. Data are shown as mean ± S.E.M, n = 5, ^**^P < 0.01 *vs.* U87 group, ^##^*P* < 0.01 *vs.* U87 + JWH133 group (one-way ANOVA followed by Tukey’s *post hoc* test).

Evidence suggests that EETs, which are products of CYP450 epoxygenases, possess mitogenic and angiogenic effects in vascular endothelial cells (33). We hypothesized that JWH133 promoted the expression of CYP2J2 in the microglia, and the EETs in M2 supernatant participated in the angiogenesis process. To verify this hypothesis, we first examined the mRNA level of CYP2J2 in the microglia and 11,12-EET concentration in microglial supernatant after JHW133 treatment. After JWH133 treatment, the mRNA level of CYP2J2 significantly increased in M2 microglia (^**^P < 0.01 *vs.* Control; **Figure 3A**), and the concentration of 11,12-EET also significantly increased in M2 microglial supernatant (^**^P < 0.01 *vs.* Control shRNA; **Figure 3B**). To investigate the effects of CYP2J2 on the JWH133-induced anti-tumor effects, lentivirus-shRNA was used to knock down the expression of CYP2J2. The results showed that lentivirus-shRNA decreased both the genetic (^**^P < 0.01 *vs.* Control shRNA; **Figure 3B**) and protein (^***^P < 0.001 *vs.* Control shRNA; **Supplementary Figure 1**) expression levels of CYP2J2. We found that CYP2J2 knockdown significantly reversed JWH133’s contribution to the increase of 11,12-EET, indicating that JWH133 promoted the expression of CYP2J2; moreover, CYP2J2 metabolized more arachidonic acid to 11,12-EET in the microglia. The addition of 11,12-EET also significantly promoted angiogenesis of HBMECs by increasing the number of tubes and branches (^**^P < 0.01 *vs.* Control; **Figures 3C–E**). Moreover, 11,12-EET treatment significantly promoted glioma growth *in vivo* (^**^P < 0.01 *vs.* U87 group; **Figures 4A, B**).

### JWH133 Treatment Inhibited Glioma Growth

Per previous finding (12), our results also showed that JWH133 treatment significantly inhibited the growth of glioma both *in vivo* (^**^P < 0.01 *vs.* U87; **Figures 4A, B**) and *in vitro* (^****^P < 0.0001 *vs.* control; **Supplementary Figure 2**). Inhibition of CB2R by AM630 could partially abolish the anti-tumor effect of JWH133 (^##^*P* < 0.01 *vs.* U87 + JWH133; **Figures 4A, B**). Those results indicated that though JWH133 promoted microglia/macrophage M2 polarization, which is conducive to the growth of glioma, the most dominating role of JWH133 in glioma treatment is attributed to its anti-tumor effects. However, administration of exogenous 11,12-EET promoted the growth of glioma *in vivo* (^**^P < 0.01 *vs.* U87; **Figures 4A, B**), but not *in vitro* (not significant *vs.* control; **Supplementary Figure 2**), indicating that 11,12-EET probably accelerates the proliferation of glioma by promoting tumor angiogenesis.

### CYP2J2 Knockdown Facilitated the Anti-Tumor Effect of JWH133 on Glioma

**Figure 5:**
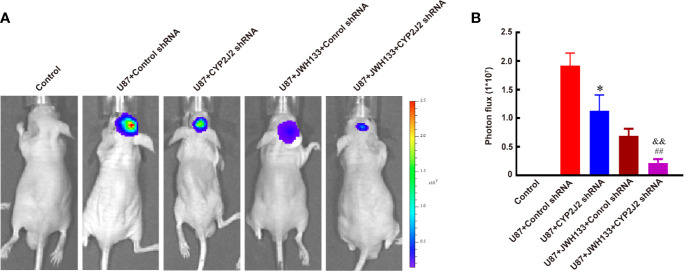
CYP2J2 knockdown facilitates the anti-tumor effect of JWH133 on glioma two weeks after implantation. Representative bioluminescent images **(A)** and the quantification **(B)** of the tumor-bearing mice implanted with U87 in the Control, U87 + Control shRNA, U87 + CYP2J2 shRNA, U87 + JWH133 + Control shRNA, and U87 + JWH133 + CYP2J2 shRNA groups [n = 5, ^*^P < 0.05 *vs.* U87 + Control shRNA group, ^##^*P* < 0.01 *vs.* U87 + JWH133 + Control shRNA group, ^&&^*P <*0.01 *vs.* U87 + CYP2J2 shRNA group (one-way ANOVA followed by Tukey’s *post hoc test*)]. Data are shown as mean ± S.E.M, n = 5, ^*^P < 0.01 *vs.* Control shRNA group, ^##^*P* < 0.01 *vs.* JWH133 + Control shRNA group, ^&&^P < 0.01 vs. U87+CYP2J2 shRNA group, (one-way ANOVA followed by Tukey’s *post hoc* Test).

Next, our results showed that CYP2J2 disruption by lentivirus-shRNA inhibited tumor growth *in vivo* (^*^P < 0.05 *vs.* U87 + Control shRNA; **Figures 5A, B**), but did not impact the growth of glioma cells *in vitro* (not significant *vs.* Control shRNA; **Supplementary Figure 3**). Interestingly, combination use of JWH133 and CYP2J2 shRNA resulted in more potently retarded tumor growth than the respective effects *in vivo* (^&&^*P* < 0.01 *vs.* U87 + CYP2J2 shRNA, ^##^*P* < 0.01 *vs.* U87 + JWH133 + Control shRNA; **Figures 5A, B**). The MRI images also supported these results (^**^P < 0.01 *vs.* U87 + CYP2J2 shRNA, ^*^P < 0.05 *vs.* U87 + JWH133; **Supplementary Figure 4**). The results of Kaplan–Meier survival curves revealed that combined administration of JWH133 and CYP2J2 shRNA significantly extended the survival of mice (**Supplementary Figure 5**).

### JWH133 Promoted the Expression of CYP2J2/EETs Through Activating ERK in Microglia

**Figure 6:**
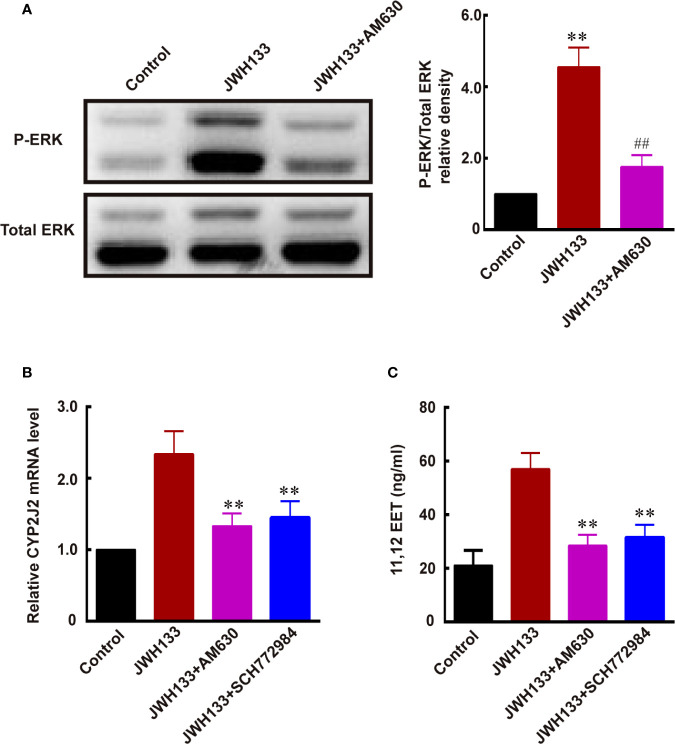
JWH133 promotes the expression of CYP2J2/11,12-EET of microglia by activating the ERK pathway. **(A)** Representative western blot images and quantitative data of p-ERK and total ERK of primary microglia in the Control, JWH133, and JWH133 + AM630 treatment groups. Data are presented as mean ± S.E.M, n = 5; ^**^P < 0.01 indicates significant differences *vs.* Control; ^##^*P* < 0.01 indicates significant differences *vs.* JWH133 treatment (one-way ANOVA followed by Tukey’s *post hoc* test). **(B)** Relative CYP2J2 mRNA level (% of Control level) in the Control, JWH133, JWH133 + AM630, and JWH133 + SCH772984 treatment groups. Data are presented as mean ± S.E.M, n = 5; ^**^P < 0.01 indicates significant differences *vs.* JWH133 treatment (one-way ANOVA followed by Tukey’s *post hoc* test). **(C)** 11,12-EET content (ng/ml) in the Control, JWH133, JWH133 + AM630, and JWH133 + SCH772984 treatment groups. Data are presented as mean ± S.E.M, n = 5; ^**^P < 0.01 indicates significant differences *vs.* JWH133 treatment (one-way ANOVA followed by Tukey’s *post hoc* test).

A previous study demonstrated that *CYP2J2* was one of the downstream target genes of ERK MAP kinases (34). Therefore, we investigated whether JWH133 promoted CYP2J2/EET expression by activating ERK. We noticed increased ERK phosphorylation after JWH133 treatment in microglia, and this JWH133-induced ERK phosphorylation was blocked by CB2R antagonist AM630 (^**^P < 0.01 *vs.* Control, ^##^*P* < 0.01 *vs.* JWH133; **Figure 6A**). The mRNA levels of CYP2J2 and concentration of 11,12-EET were both increased after JWH133 treatment. Interestingly, both CB2R antagonist AM630 and ERK antagonist SCH772984 could significantly reverse the increased level of CYP2J2 and 11,12-EET, indicating that JWH133 promoted CYP2J2/11,12-EET expression partially by activating the ERK pathway (^**^P < 0.01 *vs.* JWH133; **Figures 6B, C**).

## Discussion

Several studies have suggested that drugs that mimic the endocannabinoid system can be used to hinder or block cancer development. Two endocannabinoid receptors have been cloned *in vitro* and widely studied: cannabinoid receptor-1 (CB1R) and CB2R. Recent studies revealed that CB2R is expressed in tumor cells such as in renal cell carcinoma (35), non-small cell lung cancer (36), breast cancer (37), and gliomas (12, 38). Interestingly, local administration of the selective CB2 agonist—JWH133—or silencing CB2R expression induced a considerable regression of glioma by inducing glioma cell death (12, 38) and depressing the production of vascular endothelial growth factor (VEGF) in glioma cells (13). A previous study showed that the extent of CB2R expression was directly related to glioma malignancy. Therefore, the CB2R, which did not mediate psychoactivity, held considerable potential for anti-glioma approaches. Herein, we found that JWH133 treatment significantly inhibited the growth of glioma, indicating that the primary function of JWH133 was to inhibit glioma growth. However, importantly, JWH133 also makes a difference to other non-neoplastic cells in glioma.

Accumulating evidence indicates that microglia/TAMs promote glioma growth and invasion. Depletion of microglia by toxin clodronate (39) or a transgenic method (40) could result in reduced glioma invasion and growth *in vivo*. The mechanism mainly focused on the direct communication between microglia/TAMs and glioma. Several factors that are synthesized and released by microglia/TAMs have been reported to increase the invasion of glioblastomas or glioma, such as stress-inducible protein 1 (STI1), epidermal growth factor (EGF), IL-6, and MT1-MMP (membrane type 1-matrix metalloproteinase) (40). According to our previous result, JWH133 also promoted the microglia/TAMs M2 polarization in gliomas (10). This type of M2 phenotypic cell can facilitate the growth of glioma by promoting angiogenesis, which debilitates the anti-glioma effect of JWH133. Another previous study also showed that M2 microglia/TAMs also promoted angiogenesis to facilitate glioma growth by increasing the expression of VEGF (41). Here, we found another novel mechanism by which JWH133 treatment increased the expression of CYP2J2/11,12-EET in M2 phenotypic cells by promoting ERK phosphorylation. Moreover, addition of synthetic 11,12-EET apparently promoted angiogenesis of HBMECs and the growth of glioma. Interestingly, one study showed that EETs were a potent and selective CB2 agonist (42); thus, CB2R-ERK-CYP2J2-ETTs-CB2R might be a positive feedback loop.

CYP2J2 knockdown using shRNA enhanced the anti-glioma effect of JWH133 by abrogating the increased expression of 11,12-EET. Additionally, administration of the CB2 antagonist—AM630—abolished the anti-glioma effect of JWH133 and abrogated the activation of ERK pathway and the increased expression of CYP2J2/11,12-EET after JWH133 treatment. Treatment with the ERK antagonist—SCH772984—also abolished the increased expression of CYP2J2/11,12-EET after JWH133 treatment. These lines of evidence showed the involvement of the ERK/CYP2J2/11,12-EET singling pathway in the pro-glioma effect of M2 microglia/TAMs after JWH133 treatment, thus presenting a promising therapeutic target.

Our previous study showed that CB2 agonist JWH133 treatment promoted microglia/macrophage M2 polarization in a stroke model (10). Similarly, in this study, we showed that JWH133 also promoted microglia/TAMs M2 polarization in the glioma model. Interestingly, in the stroke model, M2 phenotypic cells promote brain recovery by inhibiting local inflammation and releasing a plethora of trophic factors. Promotion of microglial M1 to M2 phenotypic conversion provides therapeutic potential for brain injury and neurodegeneration diseases. However, in the glioma model, the M2 phenotype cells promoted glioma growth/survival by secreting proangiogenic factors and immunosuppressive cytokines; thus, converting M2 to M1 phenotype has been suggested as a potential therapeutic strategy to reduce glioma growth. Therefore, it is noteworthy that M2 phenotypic cells have beneficial or detrimental roles depending on the context of different diseases.

In conclusion, we showed that the selective CB2 agonist, JWH133, could significantly inhibit glioma growth. However, JWH133 also promoted microglia/TAMs M2 polarization in the glioma, which weakened its anti-glioma effect; therefore, the JWH133-induced M2 phenotypic cells facilitated angiogenesis by releasing 11,12-EET *via* activating the ERK/CYP2J2 pathway. Inhibiting ERK activation and silencing CYP2J2 will decrease the release of 11,12-EET from JWH133-induced M2 phenotypic cells. Combination treatment of JWH133 and CYP2J2 shRNA significantly enhanced the anti-glioma effect of JWH133. This study provides a novel mechanism and strategy for heightening the CB2R-based anti-glioma therapies. Furthermore, we need to pay close attention to the effect of anti-glioma drugs on non-neoplastic cell types in the future, which might also exert pro- or anti-glioma effect through different mechanisms.

## Data Availability Statement

The original contributions presented in the study are included in the article/**Supplementary Material**. Further inquiries can be directed to the corresponding authors.

## Ethics Statement

The animal study was reviewed and approved by Ethics Committee of the Third Military Medical University.

## Author Contributions

JL and YT designed and wrote the manuscript. XL performed statistical analysis and co-wrote the manuscript. XL, XC, and YQ performed the experiments. All co-authors edited the manuscript. All authors contributed to the article and approved the submitted version.

## Funding

This study was funded by the Third Military Medical University (Grant No. 2017XQN10).

## Conflict of Interest

The authors declare that the research was conducted in the absence of any commercial or financial relationships that could be construed as a potential conflict of interest.
