# Supplementary material for: Targeting CYP2J2 to Enhance the Anti-Glioma Efficacy of Cannabinoid Receptor 2 Stimulation by Inhibiting the Pro-Angiogenesis Function of M2 Microglia
Source: Front Oncol. 2020 Nov 27;10:574277. doi: 10.3389/fonc.2020.574277 (PMC7729163; doi:10.3389/fonc.2020.574277)
Supplement: Supplementary file 1 [file DataSheet_1.docx]

**Supplementary Figures**

**Targeting CYP2J2 to enhance the anti-glioma efficacy of cannabinoid receptor 2 stimulation by inhibiting the pro-angiogenesis function of M2 microglia**

Xuejiao Lei^1^, Xuezhu Chen^1^, Yulian Quan^1^, Yihao Tao^2^*, Junlong Li^3^*

^1^ Department of Neurosurgery and Key Laboratory of Neurotrauma, Southwest Hospital, Third Military Medical University (Army Medical University), Chongqing 400038, China.

^2^ Department of Neurosurgery, The Second Affiliated Hospital, Chongqing Medical University, Chongqing 400010, China.

^3^ Office of Scientific Research Administration, Southwest Hospital, Third Military Medical University (Army Medical University), Chongqing 400038, China.

*Contact information:

Junlong Li

Office of Scientific Research Administration, Southwest Hospital, Third Military Medical University (Army Medical University), Chongqing 400038, China.

Email address: junlong1156@163.com

Yihao Tao

Department of Neurosurgery, The Second Affiliated Hospital, Chongqing Medical University, Chongqing 400010, China.

Email address: tyheva@cqmu.edu.cn

**Supplementary Figure 1**


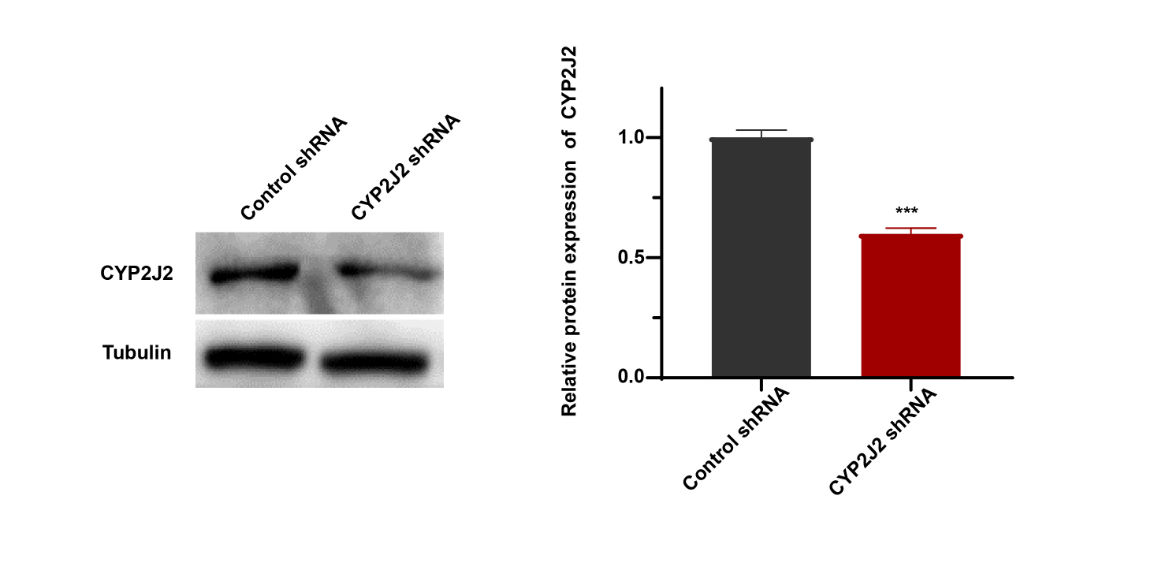


Supplementary Figure 1. **The protein expression level of CYP2J2**. Data presented mean ± S.E.M., n=5; ***P*<0.001 significantly different from Control shRNA; Student’s *t* test (two-tailed, unpaired).

**Supplementary Figure 2**


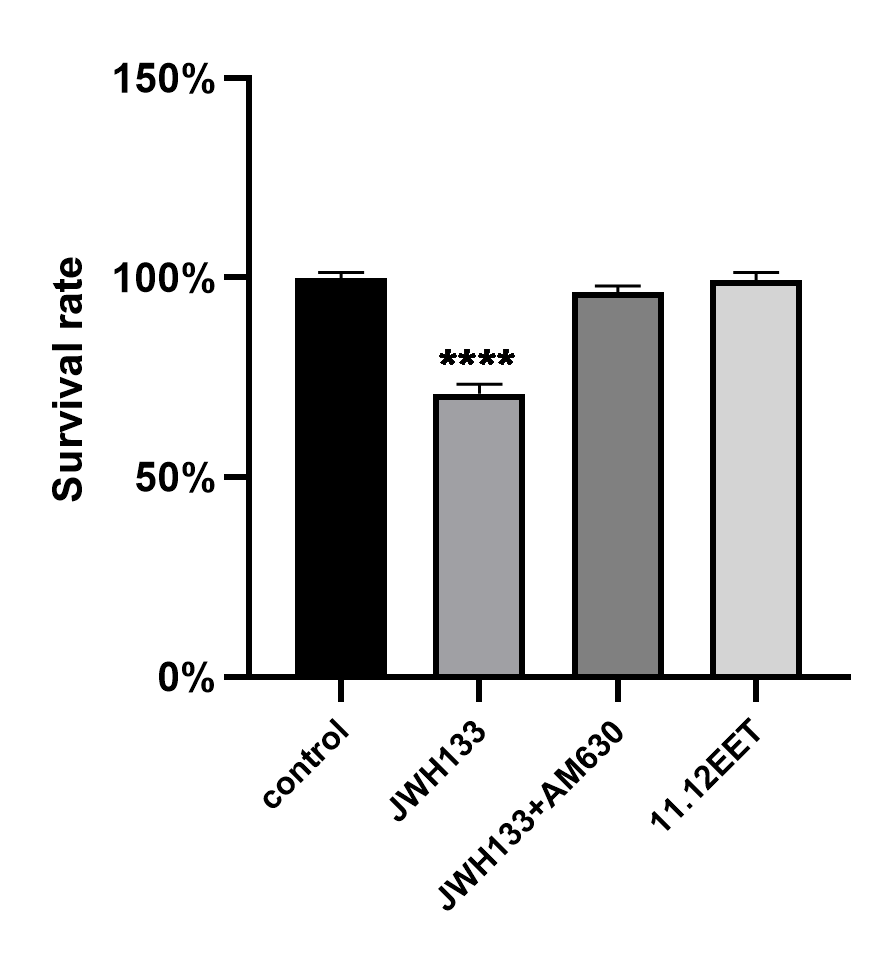


Supplementary Figure 2. **CCK8 assays were used to assess the effects of JWH133 and 11,12-EET on proliferation of glioma cells *in vitro*.** Data presented mean ± S.E.M., n=5; *****P*<0.0001 significantly different from control group, one-way ANOVA followed by Tukey’s post hoc test.

**Supplementary Figure 3**


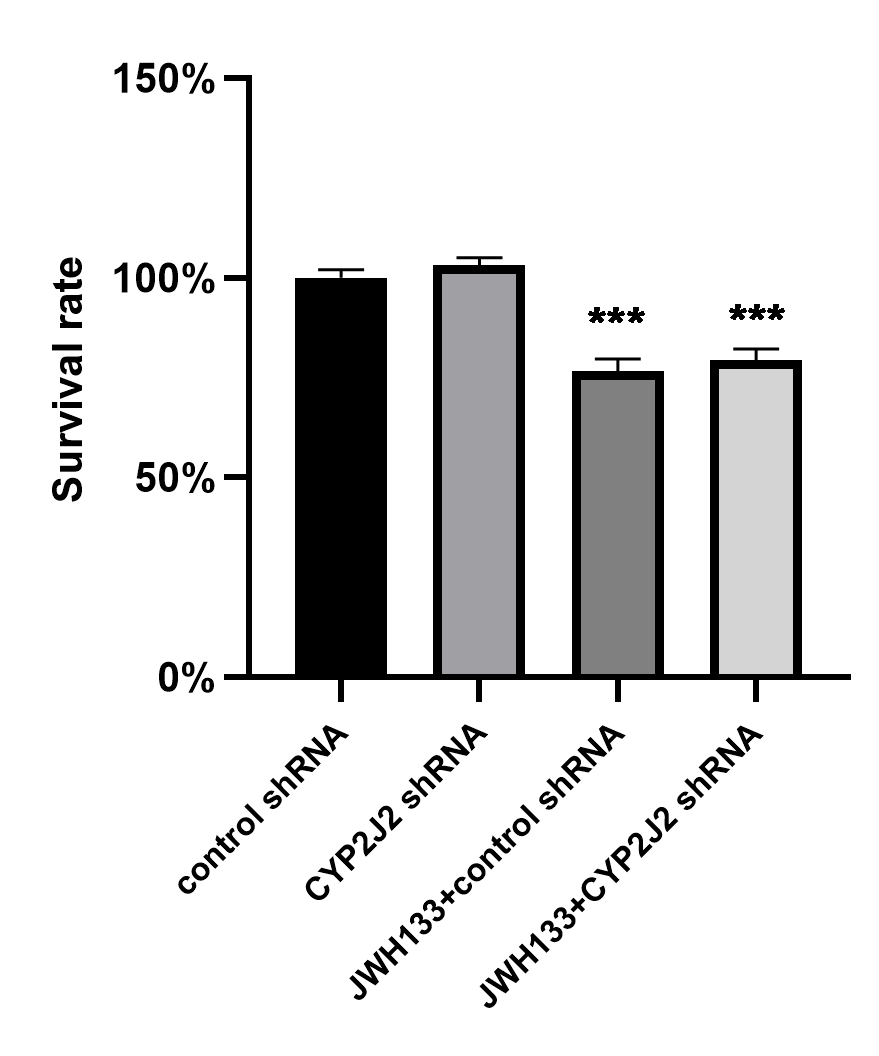


Supplementary Figure 3. **CCK8 assays were used to assess the effects of JWH133 and CYP2J2 shRNA on proliferation of glioma cells *in vitro*.** Data presented mean ± S.E.M., n=5; ****P*<0.001 significantly different from control shRNA group, one-way ANOVA followed by Tukey’s post hoc test.

**Supplementary Figure 4**


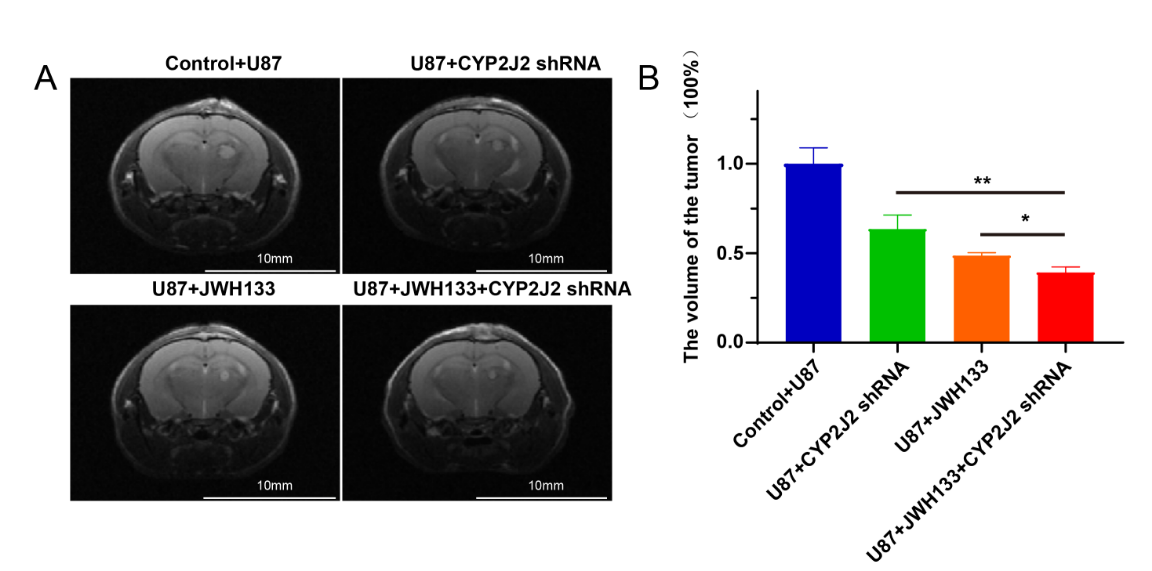


Supplementary Figure 4. **MRI images of the mice on days 14 after tumor implantation.** (A)T2-weighted imaging, (B)volume of the tumor. Values are expressed as the means ± S.E.M., n=5; ***P*<0.01, **P*<0.05, one-way ANOVA followed by Tukey’s post hoc test.

**Supplementary Figure 5**


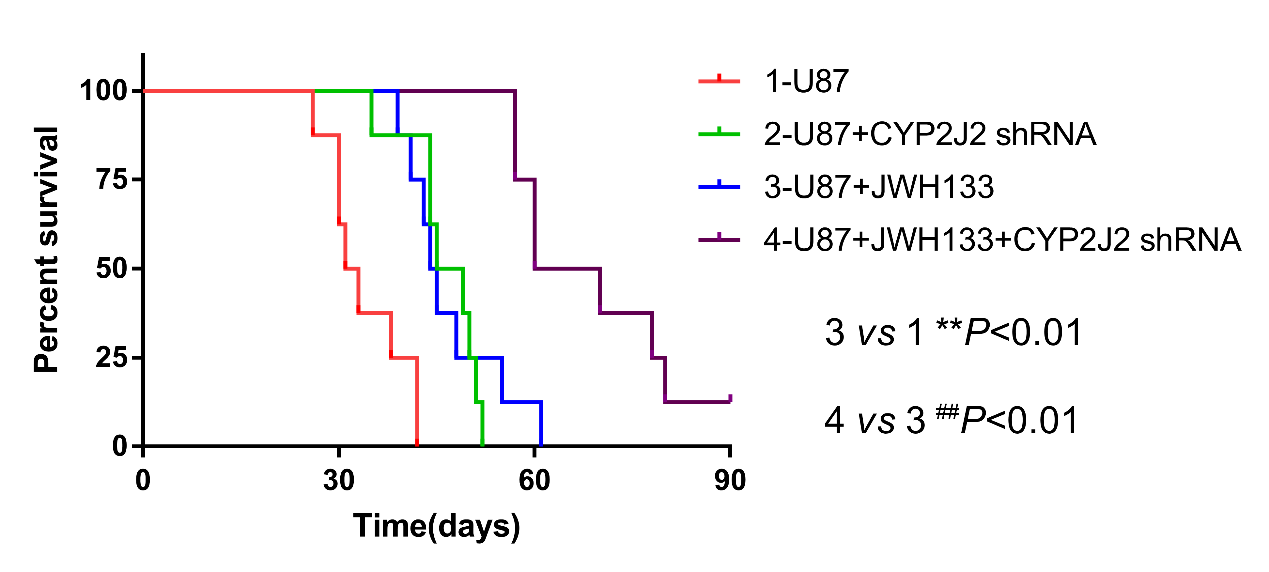


Supplementary Figure 5. **Kaplan–Meier survival curves of mouse models after corresponding treatments**. Combined treatment of JWH133 and CYP2J2 shRNA significantly prolonged the survival of mice bearing the glioma xenografts. n=8, log-rank test.
